# Training School for the Insane

**Published:** 1886-05

**Authors:** 


### TRAINING-SCHOOL FOR THE INSANE.

A new era of reform in the treatment of the insane in our public institutions has been inaugurated by the medical officers of the Buffalo State Insane Asylum. An experience of many years with this unfortunate class of patients and the management of institutions for their treatment, convinced Dr. Judson B. Andrews that much practical benefit could be derived from the systematic training of attendants. No one will deny that the trained nurses in our hospitals are much superior to the self-educated (?) Mrs. Gamps of former times, whose knowledge was supposed to be derived from experience with the sick, but which often consisted of a lot of superstitious nonsense handed down by tradition. The nurse for the insane was also educated by experience, but she always had the advantage of intelligent guidance from the physician in charge. Some who were watchful and attentive in their observations and duties became skilful attendants, but only after long years of service. In some institutions for the insane the important mental and moral qualifications, which every attendant should possess, were entirely disregarded. We well remember our experience with the muscular Pats and Bridgets, whose chief recommendation was their brawn. Such attendants so called will use physical force, and even cruelty, where a skillful nurse would, with little difficulty, manage their patients with a little persuasive kindness.—*Buffalo Medical Journal.*

**Danger From Foul Earth.**—As to the advisability of filling ravines and lots with street refuse mixed more or less with garbage, there is serious question. To a certain extent it can be safely done; but when the filling is carried up so near the desired level that only a thin shell of presentable earth can be placed on top of the rubbish, the buried filth becomes a source of danger. Being of a porous and spongy nature, it in time becomes saturated with organic matter through the leaking of sewers and vaults; and wherever a house is built on the filled premises the excavation for the cellar cuts through the crust and affords ventilation for the subterranean nastiness. That death and disease should immediately occupy new houses upon lots so filled should occasion no surprise. Recently, at Chicago, almost an entire family was swept away by malignant disease that was afterward shown to have been caused by exhalations from the cellar, the floor of which was nothing less than a spongy mass of filth, the entire lot on which the house stands having been filled with refuse from streets and backyards. This danger from promiscuous filling may serve to reconcile taxpayers to the expense of dump scows for lake work when their use shall become necessary.

